# Intraoperative linguistic performance during awake brain surgery predicts postoperative linguistic deficits

**DOI:** 10.1007/s11060-018-2863-z

**Published:** 2018-04-10

**Authors:** Wei-Han Chang, Yu-Cheng Pei, Kuo-Chen Wei, Yi-Ping Chao, Mei-Hui Chen, Heng-An Yeh, Fu-Shan Jaw, Pin-Yuan Chen

**Affiliations:** 1 Department of Physical Medicine and Rehabilitation, Chang Gung Memorial Hospital at Taoyuan, Taoyuan, Taiwan; 2 Institute of Biomedical Engineering, National Taiwan University, Taipei, Taiwan; 3 School of Medicine, Chang Gung University, Taoyuan, Taiwan; 4 Healthy Aging Research Center, Chang Gung University, Taoyuan, Taiwan; 5 Center of Vascularized Tissue Allograft, Chang Gung Memorial Hospital at Linkou, Taoyuan, Taiwan; 6 Department of Neurosurgery, Chang Gung Memorial Hospital at Linkou, Taoyuan, Taiwan; 7 Graduate Institute of Medical Mechatronics, Chang Gung University, Taoyuan, Taiwan; 8 Department of Physical Medicine and Rehabilitation, Chang Gung Memorial Hospital at Linkou, Taoyuan, Taiwan; 9 Department of Neurosurgery, Chang Gung Memorial Hospital at Keelung, No. 222, Mai-Jin Road, Keelung, 20401 Taiwan

**Keywords:** Awake craniotomy, Object naming, Semantic association, Intraoperative monitoring, Language outcome, Neuroplasticity

## Abstract

**Introduction:**

Awake craniotomy pursues a balance between extensive tumor resection and preservation of postoperative language function. A dilemma exists in patients whose tumor resection is restricted due to signs of language impairment observed during awake craniotomy. In order to determine the degree to which recovery of language function caused by tumor resection can be achieved by spontaneous neuroplasticity, the change in postoperative language function was compared to quantified intraoperative linguistic performance.

**Methods:**

The modified, short-form Boston Diagnostic Aphasia Examination (sfBDAE) was used to assess pre- and postoperative language functions; visual object naming (DO 80) and semantic-association (Pyramid and Palm Tree Test, PPTT) tests assessed intraoperative linguistic performance. DO 80 and PPTT were performed alternatively during subcortical functional monitoring while performing tumor resection and sfBDAE was assessed 1-week postoperatively.

**Results:**

Most patients with observed language impairment during awake surgery showed improved language function postoperatively. Both intraoperative DO 80 and PPTT showed significant correlation to postoperative sfBDAE domain scores (p < 0.05), with a higher correlation observed with PPTT. A linear regression model showed that only PPTT predicted the postoperative sfBDAE domain scores with the adjusted R^2^ ranging from 0.51 to 0.89 (all p < 0.01). Receiver operating characteristic analysis showed a cutoff value of PPTT that yielded a sensitivity of 80% and specificity of 100%.

**Conclusion:**

PPTT may be a feasible tool for intraoperative linguistic evaluation that can predict postoperative language outcomes. Further studies are needed to determine the extent of tumor resection that optimizes the postoperative language following neuroplasticity.

## Introduction

The main purpose of awake craniotomy for tumor resection is to sufficiently resect tumors while preserving eloquent functions for facilitating the patients’ postoperative quality of life [1–4]. Therefore, patients with brain tumors located near the eloquent areas will benefit from awake craniotomy. To identify eloquent areas, intraoperative stimulation mapping (ISM) and functional monitoring are performed after patients are awoken from general anesthesia [3, 5].

In contrast to motor and sensory function, the neural networks of language function are too complicated to be simply identified by intraoperative electrophysiological investigations and have highly individual variation in the patients with brain tumors, rendering ISM necessary in these patients [1, 6, 7].

ISM is performed by electrically stimulating the brain while the patient is performing a linguistic task [4, 5]. Specifically, a brain area is recognized as a putative language-positive site if electrical stimulation suppresses the patient’s linguistic ability [8, 9]. Before resecting the tumor, ISM is first applied to locate the eloquent area in cortex. Tumor resection then proceeds through the language-negative areas while language functional monitoring and intermittent ISM are also applied during the entire process of tumor resection [5, 10].

During awake surgery, tumor resection is finished when either extensive tumor excision is completed or when tumor excision has induced slight linguistic impairments [11]. Specifically, for patients whose tumor excision was stopped due to surgery-induced linguistic impairment (SLI), it is thought that safety margins were inadequate along the language-eloquent areas [10, 12–14], and thus postoperative language deficits may be noted and follow-up is especially needed [10–12]. In this sense, these is a conflict between the preservation of language function and the extent of tumor excision. To the best of our knowledge, no literature has yet established an explicit criterion that defines the degree of SLI that warrants the cessation of tumor resection along the eloquent areas. Also, the SLI may recover postoperatively through spontaneous neuroplasticity [10–12]. To this end, it is necessary to characterize intraoperative linguistic performance that would predict a desired postoperative language function given the existence of SLI.

In this study, patients received intraoperative functional monitoring using visual object naming and sematic-association tests, and pre- and postoperative language evaluations using the modified short-form Boston Diagnostic Aphasia Examination (sfBDAE) [9]. Specifically, their linguistic performance in the terminal stage of tumor resection was recorded. The underlying hypothesis was that intraoperative linguistic function can predict postoperative language outcomes. This study sought to determine a cutoff value for intraoperative performance that could predict a satisfactory postoperative result, given that SLI may be inevitable in some patients.

## Methods

### Subjects

All aspects of the study were approved by the Human Studies Research Committee of Chang Gung Medical Foundation, and written informed consent was obtained from each subject before recruitment.

Subjects were recruited from the neurosurgery outpatient clinic at a medical center from Dec 2015 to Nov 2017. Inclusion criteria were adults (> 20 years old) who were: (1) diagnosed with brain tumor located near the left eloquent areas; (2) could cooperate with per-, intra-, and postoperative linguistic evaluations; and (3) tolerated intraoperative functional monitoring until the terminal stage of tumor excision.

Subjects who could not completely finish the postoperative follow-up were excluded. In addition, subjects with incomplete preoperative data or intraoperative video recording were further excluded from analysis.

### Pre- and postoperative evaluation

To simply the pre- and postoperative linguistic evaluations, several subtests were modified from the Chinese version of the sfBDAE [9], which was translated and revised by speech therapists at Taipei Veterans General Hospital, Taipei, Taiwan. The selected subtests [including word discrimination, complex ideational material, repeating phrases/sentences (common and rare phrases/sentences), responsive naming, and visual confrontation naming] were used to investigate major speech dimensions, such as auditory and visual naming, comprehension, repetition, articulation, and semantics. In addition, the total time taken for patients’ response to those subtests were recorded. To simplify the procedure, only the first eight questions in the subtest of complex ideational material were applied in the evaluation. Postoperative evaluation was performed within 1 week and 3 months after surgery.

### Intraoperative linguistic tests

Number counting, i.e., Dénomination d’objet (DO) 80, was applied as a visual object naming test and Pyramids and Palm Trees Test (PPTT) was used as a semantic test of association for intraoperatively linguistic testing [15].

DO 80 is composed of 80 black-and-white pictures with proportional categories and the patient is required to name every picture within 4 s. For each trial, failure is counted when the patient shows anomia, speech arrest, misnaming, dysarthria, or delayed naming.

In PPTT,[16] three black-and-white pictures are presented every time with one picture on the top and the others on the bottom. Without time restriction, the patients select one out of two pictures on the bottom which is mostly related to the picture on the top, and then names the top and selected pictures. For each trial, failure is counted when the patient shows anomia, speech arrest, misnaming, dysarthria, miscorrelation of two pictures, or delayed responses compared with initially intraoperative testing. As described above, PPTT includes not only picture naming but also retrieval of comprehensive sematic information and semantic similarity judgment which recruit more language function than visual object naming tests [17]. It had been simply applied to awake craniotomy for mapping inferior fronto-occipital fasciculus, an important fasciculus of language connectivity [18]. Language is a complex function, so the more complex but simply-applied test was used to monitor language function during tumor resection.

Patients were evaluated by DO 80 and PPTT before surgery. For the intraoperative evaluation, those pictures which patients failed to name or to which they replied incorrectly were removed and the rest of pictures were preserved.

The scores of the intraoperative linguistic tasks (DO 80 and PPPT) were determined using the accuracy rate which was defined by the proportion of pictures to which the patient responded correctly. All intraoperative testing was performed by a physiatrist or a neurologist.

### ISM procedure

After each patient awoke, number counting combined with continuous movement of the upper limb were first applied to determine the optimal intensity of ISM by electrical stimulation of the primary motor area to verify the minimal intensity that elicited observed motor responses of the contralateral face [18]. Specifically, a bipolar electrode with 5-mm spaced tips was applied to deliver biphasic current stimulation (pulse frequency, 50 Hz; single pulse phase duration, 1 ms; amplitude, 2–10 mA) (Model OCS2 Ojemann Cortical Stimulator, Integra LifeSciences Corporation, Saint Priest, France).

The intensity of electrical stimulation was increased gradually until oral twitching, speech arrest, dysarthria, or the motor response of a limb was observed, and electrical stimulation at this magnitude of current was used for subsequent stimulation. During cortical ISM for language-area mapping, DO 80 (visual object naming test) was applied to identify the language-positive and language-negative sites. The patient named each picture displayed on the video display within 4 s while each regional cortex was subsequently suppressed for less than 4 s by the electrical current. When the naming errors appeared and the abnormality in naming was confirmed in two consecutive tests, the electrically-suppressed area was recognized as a putative language-associated (-positive) area. When tumor excision started, the patient continuously and alternatively performed DO 80 and PPTT for language functional monitoring. When the patient revealed naming errors in DO 80, or incorrect responses in PPTT, the neurosurgeon was alerted and tumor excision was stopped to preserve the patient’s speech function.

### Statistical analysis

The scores (accuracy rates) of DO 80 and PPTT represent intraoperative linguistic evaluations, and the total and subtest scores of sfBDAE (in the postoperative setting) represent postoperative linguistic outcomes. Pearson’s correlation was used to analyze the correlations between data obtained in intraoperative and postoperative linguistic evaluations. Using the results of postoperative evaluations as the dependent variables, stepwise linear regressions were performed to selectively predict factors, including the DO 80 and PPTT. Receiver operating characteristic (ROC) analysis was further used to distinguish the discrimination ability and the optimal cutoff value when intraoperative linguistic evaluations were used to predict postoperative language outcomes. The areas under curve (AUC) and Youden index were used to judge the discrimination ability.

## Results

**Fig. 1:**
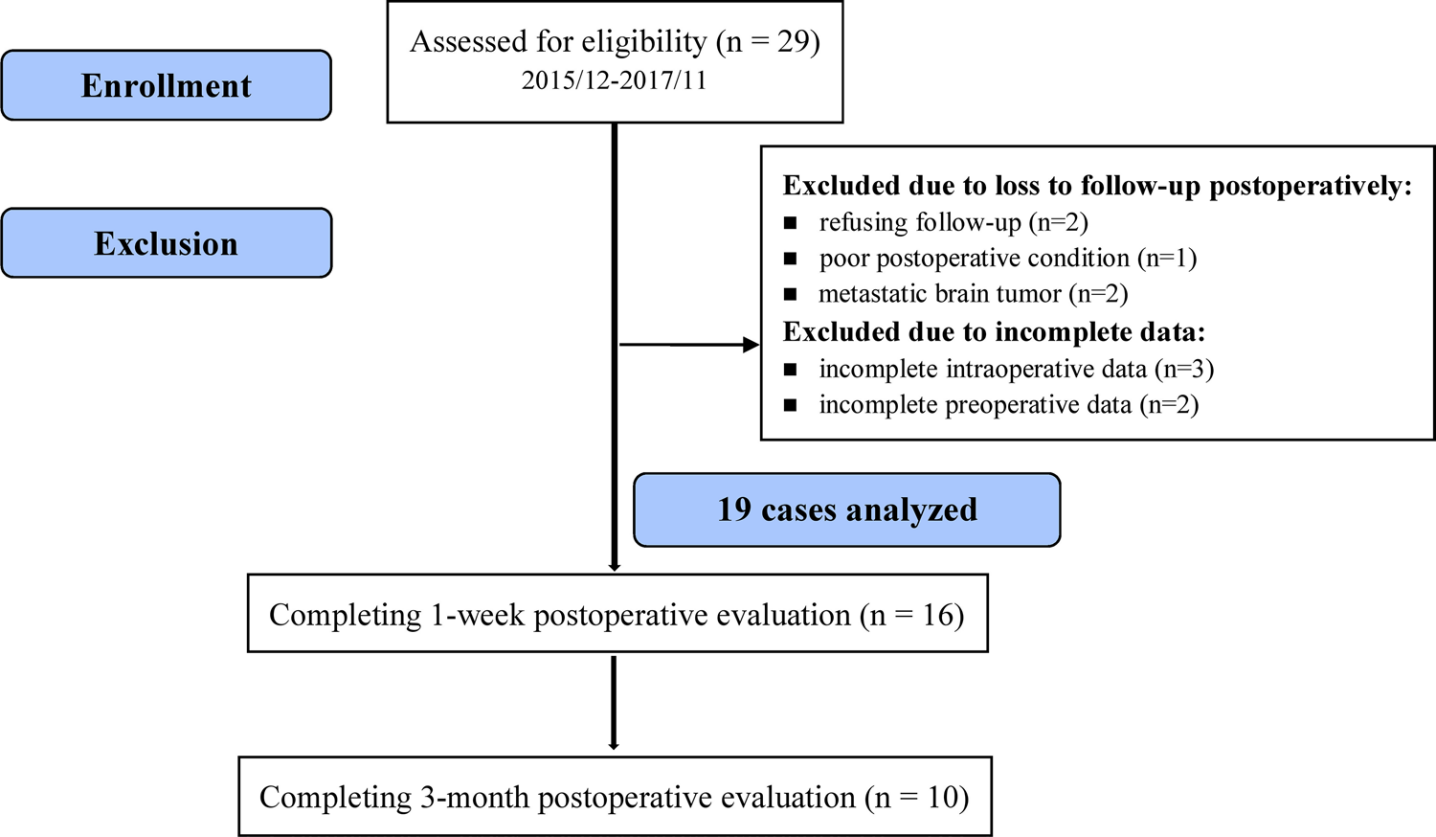
Flowchart for subject enrollment, exclusion, and analysis

Twenty-nine patients with brain tumors located near the left eloquent areas were recruited from Dec 2015 to Nov 2017 (Fig. 1).

Among these patients, ten were excluded for metastatic brain tumor confirmed by pathologic reports (n = 2), refusal of postoperative evaluation (n = 2), poor medical condition (n = 1), incomplete preoperative evaluation data (n = 2) and incomplete intraoperative evaluation (n = 3). The remaining 19 patients were analyzed, including 16 patients who completed 1-week postoperative evaluation and 10 patients who completed 3-month postoperative evaluation.

Demographic data are shown in Table 1. The percentage of high grade brain tumor (grade III and IV) was 53%. Eleven patients (58%) received total resection (> 95%) [5], and three (16%) received subtotal resection (95–85%). The brain tumors were most commonly located in the frontal (64%) and frontotemporal lobes (16%). Ten patients (52%) were intraoperatively detected language-positive sites which mainly located in left middle and inferior frontal gyri (42%). In immediate postoperative MRI, one patient (5%) had acute infarction at left insula and twelve patients (63%) had minimal perioperative infarction and vasogenic edema surrounding surgical cavity. The patient with left insular infarction had immediate decline in language function (the total score of sfBDAE declining from 134 to 101) but low scores of the intraoperative linguistic tasks (DO 80: 0.86 and PPPT: 0) was also noted. In contract, the patients with minimal infarction or vasogenic edema had variable changes in pre- and postoperative language function.

**Table 1:** Demographics and resection rate, pathology, and location of tumors

	n (%) or mean ± std
Sex (male/female)	9/10
Age (years)	41.0 ± 11.5 (20–65)
Resection rate	44.3–100%
>95%	11 (58)
85–95%	3 (16)
<85%	5 (26)
Histologic and genetic results^a^	
Glioblastoma (grade IV)	
IDH-wild-type	1 (5)
IDH-mutant	1 (5)
Anaplastic astrocytoma (grade III)	
IDH-wild-type	1 (5)
NOS	3 (16)
Anaplastic oligoastrocytoma, NOS (grade III)	3(16)
Anaplastic oligodendroglioma, 1p/19q-codeleted (grade III)	1 (5)
Diffuse astrocytoma, IDH-mutant (grade II)	3(16)
Oligodendroglioma (grade II)	
IDH mutant	1 (5)
NOS	3 (16)
Arteriovenous malformation	1 (5)
Chronic inflammation	1 (5)
Location	
Frontal lobe	12 (64)
Parietal lobe	1 (5)
Frontotemporal	3 (16)
Frontoparietal	1 (5)
Fronto-insula	1 (5)
Insula	1 (5)
Mapping results	
Pars triangularis	3 (16)
Pars opercularis	3 (16)
Posterior-middle frontal gyrus	2 (10)
Inferior parietal gyrus	2 (10)
Negative	9 (48)
Immediate postoperative MRI findings	
Perioperative infarction at left insula	1 (5)
Decline of language function	1
Perioperative infarction surrounding the surgical cavity	4 (21)
No change or improvement of language function	3
Decline of language function	1
Vasogenic edema surrounding the surgical cavity	8 (42)
No change or improvement of language function	6
Decline of language function	2
Negative	6 (32)

*n* number, *std* standard deviation

^**a**^Classification referring to The 2016 World Health Organization Classification of Tumors of the Central Nervous System

### Correlation between intraoperative and postoperative evaluations

At 1-week follow-up, the accuracy of DO 80 significantly correlated with the postoperative total sfBDAE and subtest sfBDAE scores, except the subtest of duration of complex ideational material (Table 2).

**Table 2:** Correlation between evaluations in the terminal stage of tumor excision and the 1-week postoperative sfBDAE

Postoperative sfBDAE	Intraoperative evaluation			
	DO 80		PPTT	
	*r*	p value	*r*	p value
Total score	0.68**	< 0.01	0.78**	< 0.01
Subtest score				
Word discrimination	0.68**	< 0.01	0.78**	< 0.01
Duration of word discrimination	− 0.86**	< 0.01	− 0.79**	< 0.01
Complex ideational material	0.67**	< 0.01	0.85**	< 0.01
Duration of complex ideational material	− 0.17	0.52	− 0.43	0.10
Repeating common phrases/sentences	0.59*	< 0.05	0.87**	< 0.01
Repeating seldom phrases/sentences	0.72**	< 0.01	0.95**	< 0.01
Duration of repeating phrases/sentences	− 0.64**	< 0.01	− 0.83**	< 0.01
Responsive naming	0.66**	< 0.01	0.75**	< 0.01
Duration of responsive naming	− 0.61*	< 0.05	− 0.81**	< 0.01
Visual confrontation naming	− 0.67**	< 0.01	0.73**	< 0.01
Duration of visual confrontation naming	− 0.73**	< 0.01	− 0.74**	< 0.01

*DO 80* Dénomination d’objet 80, *PPTT* pyramid and palm tree test, *sfBDAE* modified short form Boston Diagnostic Aphasia Examination, *r* correlation coefficient

∗p < 0.05, ∗∗p < 0.01

Among them, the sfBDAE subtests of word discrimination, duration of word discrimination, complex ideational material, repeating seldom phrases/sentences, repeating common phrases/sentences, duration of repeating phrases/sentences, responsive naming, duration of responsive naming, visual confrontation naming and duration of visual confrontation naming showed significant correlation with DO 80 (p < 0.01). The correlation coefficient in DO 80 ranged from 0.59 to 0.73.

Analogous correlation was demonstrated in PPTT. The correlation coefficient ranged from 0.73 to 0.95. Except duration of word discrimination, most sfBDAE subtests had higher correlation coefficients with the PPTT than with DO 80. The correlation coefficient between the accuracy rates of DO 80 and PPTT was 0.82 (p < 0.01), indicating the DO 80 and PPTT also correlated.

In the 3-month follow-up, the DO 80 or PPTT did not significantly correlate with the postoperative total sfBDAE or subtest sfBDAE scores. The overall correlation coefficient ranged from 0.01 to 0.47 (the range of p value: 0.17–0.99).

### The explanatory power of accuracy rates to postoperative sfBDAE scores

As DO 80 and PPTT covariate with each other, it was necessary to perform stepwise multiple linear regression to select the parameters that best predicted postoperative sfBDAE scores in 1-week follow-up (Table 3) and this analysis was not performed for 3-month post-op as the aforementioned correlation was not significant. The results showed that PPTT predicted total sfBDAE and the other sfBDAE subtest scores, except for the BDAE subtest of duration of word discrimination for which DO80 was selected. The explanatory power (adjusted R^2^) of PPTT ranged 0.51–0.89 (all p < 0.01) and the adjusted R^2^ of DO 80 to duration of word discrimination was 0.73 (p < 0.01). These results demonstrated that PPTT was the most suitable predictor of 1-week postoperative sfBDAE scores.

**Table 3:** Stepwise multiple linear regression for predicting 1-week postoperative sfBDAE scores

Postoperative sfBDAE	Predictors					
	DO 80		PPTT		Selected model	
	SCoe	p value	SCoe	p value	Adjusted R^2^	p value
Total score			0.78**	< 0.01	0.58**	< 0.01
Subtest score						
Word discrimination			0.78**	< 0.01	0.58**	< 0.01
Duration of word discrimination	− 0.86**	< 0.01			0.73**	< 0.01
Complex ideational material			0.85	< 0.01	0.71**	< 0.01
Repeating common phrases/sentences			0.87**	< 0.01	0.73**	< 0.01
Repeating seldom phrases/sentences			0.95**	< 0.01	0.89**	< 0.01
Duration of repeating phrases/sentences			− 0.83**	< 0.01	0.67**	< 0.01
Responsive naming			0.75**	< 0.01	0.53**	< 0.01
Duration of responsive naming			− 0.81**	< 0.01	0.63**	< 0.01
Visual confrontation naming			0.73**	< 0.01	0.51**	< 0.01
Duration of visual confrontation naming			− 0.74**	< 0.01	0.51**	< 0.01

*DO 80* Dénomination d’objet 80, *PPTT* pyramid and palm tree test, *sfBDAE* modified short form Boston Diagnostic Aphasia Examination, *SCoe* standardized regression coefficient

∗p < 0.05, ∗∗p < 0.01

### ROC analysis for identifying the ability and cutoff value

**Fig. 2:**
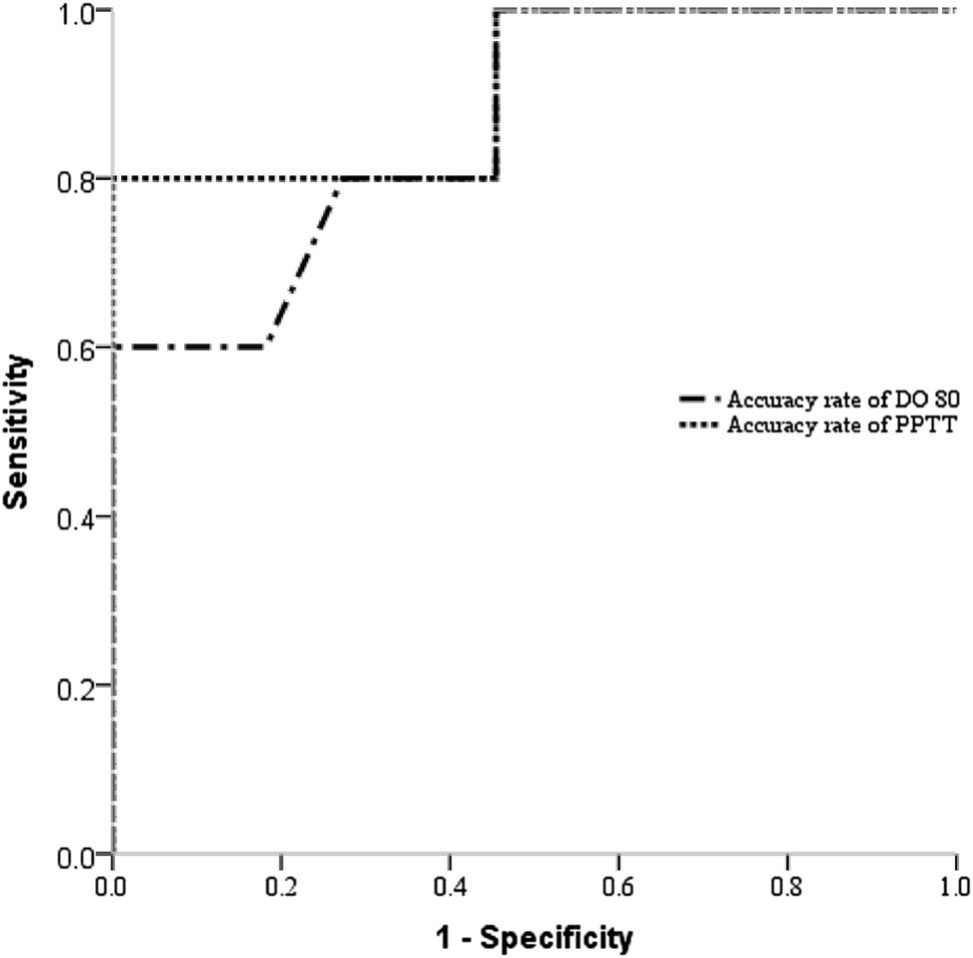
ROC analysis of discriminatory ability of DO 80 versus PPTT in predicting language deficits at 1-week follow-up

ROC analysis was performed to identify the discriminatory ability and cutoff value of DO 80 and PPTT for predicting postoperative language deficits. The state variable was defined as whether postoperative total sfBDAE score at 1-week follow-up was less than its preoperative score. The ROC curves in Fig. 2 showed the PPTT and D80 had an AUC of 0.909 (p = 0.011) and 0.864 (p = 0.023), respectively, indicating a superior discrimination performance in PPTT.

Choosing a cutoff value of 0.75, PPTT has a sensitivity of 80% and specificity of 100%. Choosing a cutoff value of 0.89, DO 80 has a sensitivity of 60% and the specificity of 100%. Again, PPTT showed superior sensitivity and specificity compared with DO 80 in predicting postoperative language deficits.

## Discussion

To the best of our knowledge, this study is the first to characterize the discriminatory ability of intraoperative linguistic evaluation in predicting postoperative language deficits. Intraoperative linguistic tasks should be kept simple as the electrical stimulation of ISM usually last less than 4 s, a parameter that was chosen to avoid electrically elicited intraoperative seizure [4, 7, 19]. During tumor resection and subcortical functional monitoring, through simple linguistic testing using DO 80 or PPTT, the neurological expert can subjectively judge whether intraoperative linguistic impairment was induced by surgical injury to language areas versus the patient’s fatigue or inattention when the patient was giving incorrect responses [4, 11, 20].

Some researchers have proposed that intermittent ISM can also confirm the SLI [11, 20–22]. If linguistic errors are confirmed by consistent linguistic impairment through several electrical stimulations, it is believed that no margin around the language-association areas is left and further tumor excision will cause permanent postoperative language deficits [11, 21]. However, this strategy has not been generally accepted. Talacchi et al. stated that further research is required to validate this procedure and Berger et al. proposed that a safety margin of 7–10 mm from language areas was necessary to prevent permanent language deficits [11, 14, 23, 24]. Rather than totally depending on the neurological assessors’ expertise, an end point is needed to determine the cutoff value of intraoperative evaluations to avoid more extensive tumor excision along the eloquent area.

Based on correlation and stepwise linear regression analyses, we found the PPTT was superior to DO 80 in predicting language performance at 1-week follow-up. Visual object naming, such as DO 80, that involves both semantic and phonologic processing [4] is the most commonly used intraoperative linguistic task for cortical ISM and subcortical functional monitoring [4, 11, 18, 25] because anomia is an almost universal presentation of aphasia and picture naming detects a large distribution of putative language areas [8, 15, 26]. Three epicenters, including the occipital lobe for visual perception, left posterior temporal areas for semantic retrieval, and left inferior frontal cortex for semantic execution and phonological production are involved in visual object naming. Furthermore, inferior fronto-occipital fasciculi interconnect these epicenters [13, 27–29]. Visual object naming tests involve speech comprehension and production areas, and the fasciculi interconnecting them, making it suitable to identify language-associated areas.

PPTT, which is a more sensitive predictor of postoperative language deficits, involves analogous speech processing, but requires more complicated semantic processing, such as semantic similarity judgment, and inextricable phonological processing [13, 17, 30]. In contrast to object naming, PPTT involves retrieval of both morphological and corresponding semantic information when identifying the semantic association between the top and bottom pictures. In addition, it requires the patient to articulate the names of the two selected pictures, which thus recruits more cognitive processes. Consequently, fasciculi serving connectivity of language and other cognitive functions may be at risk of damage during tumor excision. Given that language involves not only classical speech areas but also a representation of complicated cognitive functions [31, 32], we proposed that the linguistic tasks that involve more widespread cortical and subcortical processing mechanisms, such as PPTT, might be more suitable for predicting permanent language deficits.

ROC analysis in this study showed that PPTT can effectively predict postoperative language deficits. We chose a cutoff value of accuracy rate of 0.75 as it had reasonable sensitivity and specificity and was practical to use (as 0.75 indicates at least one error in every four consecutive trials).

Few researchers have yet defined the intraoperative linguistic impairment that warrants stopping tumor resection and which can be compensated by postoperative neuroplasticity. Duffau et al. reported that tumor resection should stop if mild linguistic disturbance was confirmed by electrically stimulating the edge of the surgical cavity [11, 20, 22, 33] but this strategy cannot verify the limit of postoperative neuroplasticity. According to our results, tumor resection should be slowed and carefully moved forward when the accuracy rate of PPTT begins to reduce. If the accuracy rate of PPTT closely drops to the cutoff value of 0.75, tumor resection should be stopped to prevent immediate postoperative language deficits.

The difference between DO 80 and PPTT in discriminatory ability may also be accounted for by their task structure. Specifically, DO 80 and PPTT had different time limits for verifying correct response. Each picture of DO 80 was shown every 4 s and correct naming was confirmed even though delayed naming compared with previous responses without exceeding 4 s. In contrast, PPTT response required no time restriction and delayed response was directly compared with previous testing according to a neurological expert’s judgment. To this end, we hypothesized that moving the time limits for DO 80 or including the time limits for PPTT when applied intraoperatively might dramatically alter their discriminatory ability. Indeed, a researcher found that delayed speaking speed will affect the patient’s return-to-work change, making it legitimate to monitor the duration of patient’s response during intraoperative evaluations [34].

In addition to intraoperative language performance, many confounding factors, including preoperative language status, subcortical language tract identified, language-positive sites within the tumor, intraoperative complications and non-frontal lesion location had been reported to predict postoperative language performance [1, 19, 35, 36]. Besides, immediately postoperative infarction or intracranial hemorrhage involving language areas should be considered as a risk factor of postoperative language deficits [11, 37]. Actually, we did analyze the correlation between postoperative language outcomes and preoperative language status, language-positive sites mapped, tumor location, intraoperative language performance at terminal stage of tumor resection and postoperative stroke noted in immediate MRI. However, we only found preoperative language status and intraoperative language performance had significant correlation with language outcomes. The difference of our findings to aforementioned studies may be accounted for by small sample size in our study. For the same reason, it is difficult to perform subgroup analysis to reduce the confounding factor of preoperative language status.

This study had several limitations. First, inadequate sample sizes influenced the power to yield precise accuracy rates of intraoperative tasks and long-term language outcomes. Second, this study only analyzed the patient’s performance at the terminal stage of tumor resection rather than after tumor resection had stopped. The short period between continued tumor resection and coagulation process may have also affected the accuracy rate, especially in cases with new onset SLI. Third, based on our previous work, including other risk factors (such as preoperatively sfBDAE total score) will likely improve the predictive ability of the postoperative sfBDAE subtest scores. To this end, a future study using subgroup analysis with aphasia and non-aphasia groups will be performed to improve the prediction model.

## Conclusion

PPTT may be a feasible tool for intraoperative linguistic evaluation that can predict postoperative language outcomes. If the accuracy rate of PPTT approaches the cutoff value of 0.75, tumor resection should be stopped to prevent immediate postoperative language deficits. Further studies are needed to determine the extent of tumor resection that optimizes postoperative language given that SLI may recover postoperatively through spontaneous neuroplasticity.
